# Molecular Cloning and Functional Analysis of Gene Clusters for the Biosynthesis of Indole-Diterpenes in *Penicillium crustosum* and *P. janthinellum*

**DOI:** 10.3390/toxins7082701

**Published:** 2015-07-23

**Authors:** Matthew J. Nicholson, Carla J. Eaton, Cornelia Stärkel, Brian A. Tapper, Murray P. Cox, Barry Scott

**Affiliations:** 1 Institute of Fundamental Sciences, Massey University, Private Bag 11 222, Palmerston North 4442, New Zealand; E-Mails: mattnicholson.nz@gmail.com (M.J.N.); c.j.eaton@massey.ac.nz (C.J.E.); conni.staerkel@gmail.com (C.S.); m.p.cox@massey.ac.nz (M.P.C.); 2 AgResearch, Grasslands Research Centre, Private Bag 11 008, Palmerston North 4442, New Zealand; E-Mail: brian.tapper@agresearch.co.nz

**Keywords:** indole-diterpene, penitrems, shearinines, janthitremanes, gene clusters

## Abstract

The penitremane and janthitremane families of indole-diterpenes are abundant natural products synthesized by *Penicillium crustosum* and *P. janthinellum*. Using a combination of PCR, cosmid library screening, and Illumina sequencing we have identified gene clusters encoding enzymes for the synthesis of these compounds. Targeted deletion of *penP* in *P. crustosum* abolished the synthesis of penitrems A, B, D, E, and F, and led to accumulation of paspaline, a key intermediate for paxilline biosynthesis in *P. paxilli*. Similarly, deletion of *janP* and *janD* in *P. janthinellum* abolished the synthesis of prenyl-elaborated indole-diterpenes, and led to accumulation in the latter of 13-desoxypaxilline, a key intermediate for the synthesis of the structurally related aflatremanes synthesized by *Aspergillus flavus*. This study helps resolve the genetic basis for the complexity of indole-diterpene natural products found within the *Penicillium* and *Aspergillus* species. All indole-diterpene gene clusters identified to date have a core set of genes for the synthesis of paspaline and a suite of genes encoding multi-functional cytochrome P450 monooxygenases, FAD dependent monooxygenases, and prenyl transferases that catalyse various regio- and stereo- specific oxidations that give rise to the diversity of indole-diterpene products synthesized by this group of fungi.

## 1. Introduction

Indole-diterpenes are a large structurally diverse group of natural products, many of which are potent tremorgenic mammalian mycotoxins [1,2,3]. This group of metabolites appears to be confined to a limited number of filamentous fungi within the Eurotiomycetes (e.g., *Penicillium* and *Aspergillus* spp.) and Sordariomycetes (e.g., *Epichloë*, *Albophoma* and *Nodulisporium* spp.) [4]. Indole-diterpenes have a number of biological activities including insect feeding deterrence [5,6], modulation of insect and mammalian potassium ion channels [7,8], and inhibition of specific enzymes [9]. These diverse biological activities have made this group of compounds particularly attractive as potentially new bioactive and therapeutic agents.

**Figure 1:**
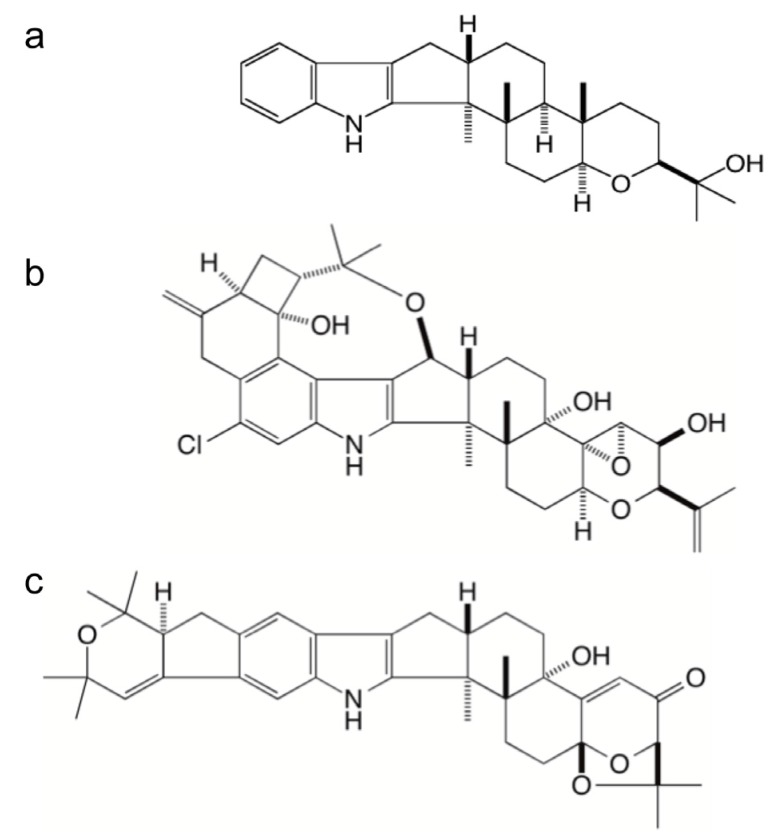
Chemical structures of paspaline (**a**), penitrem A (**b**), and shearinine A (**c**).

Using *Penicillium paxilli* as a model experimental system we have identified and functionally characterized the genes required for the synthesis of paxilline [10,11], a potent inhibitor of calcium activated BK channels [7]. Genetic analysis of *P. paxilli* has established that a cluster of seven genes is required for paxilline biosynthesis [10,11,12]. Using a *P. paxilli* mutant deleted for the entire *pax* gene cluster we showed by gene reconstitution experiments that just four of these genes, *paxG*, *paxM*, *paxB*, and *paxC*, are required for the synthesis of paspaline [13], the first cyclic indole-diterpene intermediate in this pathway (Figure 1). Based on this study we proposed a biosynthetic scheme for paspaline biosynthesis [13]. This scheme has recently been experimentally validated by reconstitution of the pathway in the heterologous host *A. oryzae* [14]. Increased chemical complexity is achieved through enzyme-specific decorations of this core structure through the action of two cytochrome P450 monooxygenases, PaxP, and PaxQ [12,15]. These additional steps have also been experimentally validated by reconstitution of paxilline biosynthesis in the heterologous host *A. oryzae* [14].

A comparative analysis of related indole-diterpene gene clusters isolated from *Epichloë festucae* [16,17], a symbiont of forage grasses, and *Aspergillus flavus* [18,19], has confirmed that these organisms also have the core set of indole-diterpene biosynthetic genes as well as unique genes that are predicted to encode enzyme functions that catalyze the specific chemical decorations that define the predominant indole-diterpene products synthesized by these fungi *i.e.*, lolitrems (lolitremanes) and aflatrems (aflatremanes), respectively [20].

Two additional important classes of indole-diterpenes are the penitremanes and janthitremanes [20], both of which contain compounds that are potent mycotoxins [21,22,23,24] (Figure 1). The objective of this study was to clone and analyse gene clusters for indole-diterpene synthesis from strains of *P. crustosum* and *P. janthinellum*, which synthesize penitremanes and janthitremanes [25], respectively.

## 2. Results

### 2.1. Identification of Gene Clusters for Indole-Diterpene Biosynthesis in P. crustosum and P. janthinellum

**Figure 2:**
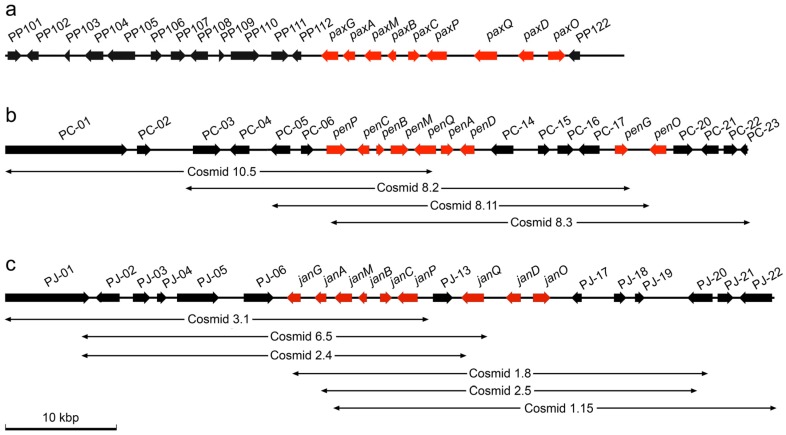
Physical maps of the *PAX* (paxilline) locus in *P. paxilli* (**a**), *PEN* (penitremane) locus in *P. crustosum* (**b**) and the *JAN* (janthitremane) locus in *P. janthinellum* (**c**). For each locus the top line represents the entire sequence and subsequent lines represent unique cosmid clones that were identified for each genome. The positions and transcriptional orientations of identified genes are indicated by arrows on the sequences with genes homologous to paxilline and post-paxilline biosynthesis in *P. paxilli* shown in red.

A preliminary analysis of chemical extracts from *P. crustosum* isolate PN2402 and *P. janthinellum* isolate PN2408 identified compounds with mass and fragmentation profiles consistent with penetremanes and janthitremanes respectively suggesting that these strains were suitable candidates for identification and characterisation of the genes responsible for indole-diterpene biosynthesis. A combination of degenerate and species-specific primers shown in Table A1 were used to amplify a variety of putative indole-diterpene cluster fragments from both species. Degenerate primers were designed to conserved regions of indole-diterpene biosynthetic genes homologous to *paxC* and *paxP* (Figure 2) after comparing homologous genes from *P. paxilli*, *A. flavus,* and *E. festucae* var. *lolii* (*=Neotyphodium lolii*) [26]. Species specific primers were subsequently designed based on the sequence of amplification products from degenerate PCR.

Successful amplification was achieved for both species using degenerate primers conC1 and conC2 resulting in putative gene fragments that were similar to the corresponding region of the *P. paxilli paxC* gene. These amplification products were 529-bp for *P. crustosum* and 535-bp for *P. janthinellum*. For *P. crustosum*, the *paxC*-like fragment was extended using primers 2402F1 (specific for the conC1-conC2 product) and degenerate primer PBR2 to produce a contiguous sequence of 2020-bp, which included 813-bp that was similar to the 3' end of *paxC*, an intergenic region of 865-bp, and 342-bp that was similar to the 3' end of *paxP* from *P. paxilli*. For *P. janthinellum* a second product of 601-bp was amplified using degenerate primers PPF1 and PPR2 that was similar to a fragment of *paxP* from *P. paxilli*. These putative C and P gene PCR products were then linked using species-specific primers 2408F1 and 2408PF1 resulting in a contiguous sequence of 2025-bp for the three overlapping amplification products which included 829-bp that was similar to the 3′ end of *paxC*, an intergenic region of 392-bp and 804-bp that was similar to the 3′ end of the *paxP*-like gene.

In both species the occurrence of closely linked putative indole-diterpene biosynthesis genes suggested that they were fragments of gene clusters for indole-diterpene biosynthesis and these fragments were therefore used to create radioactively labeled hybridization probes for screening cosmid-based genomic libraries from the respective species. For *P. crustosum* a library containing an estimated 30,000 unique clones yielded five positively-hybridizing colonies that PCR and restriction analysis demonstrated contained four non-identical overlapping cosmid clones as shown in Figure 2a. For *P. janthinellum* a library containing an estimated 50,000 unique clones yielded 16 positively-hybridizing colonies that PCR and restriction analysis demonstrated contained six non-identical overlapping cosmid clones as shown in Figure 2b. For both species two clones were selected for sequencing with minimum overlap, thus maximizing the sequence information that was obtained.

Cosmid 10.5 and Cosmid 8.3 were sequenced for *P. crustosum* to generate a contiguous sequence of 66,022-bp. For *P. janthinellum* Cosmid 3.1 and Cosmid 1.15 were sequenced generating a 68,344-bp contig. Bioinformatic analysis of both contig sequences identified putative genes as shown in the physical maps in Figure 2. Predicted functions for gene products based on similarity with characterized proteins are detailed in Table 1 (*P. crustosum*) and Table 2 (*P. janthinellum*). Using the same naming convention as for other indole-diterpene gene clusters we have designated these the *PEN* (penitremane) and *JAN* (janthitremane) gene clusters with putative indole-diterpene biosynthetic genes designated *pen* and *jan* for *P. crustosum* and *P. janthinellum*, respectively. This nomenclature follows the same convention as for *pax* genes in the *PAX* cluster such that the homologs of *paxC*, for example, have been designated *penC* for *P. crustosum* and *janC* for *P. janthinellum*.

**Table 1:** Known and predicted functions of genes at the *P. crustosum* *PEN* locus.

Gene/ORF	No. of Exons	Predicted Product Size (aa)	Predicted Function	Top BLASTp Match		
				Organism	*E*-Value	Accession No.
PC-01 (partial)	≥5	≥3555	NRPS (HC-toxin synthase)	*Penicillium digitatum*	0.0	EKV04333
PC-02	1	445	Hypothetical	*Aspergillus oryzae* RIB40	1e^−166^	XP_003189837
PC-03	2	743	Hypothetical cell wall protein	*P. digitatum*	0.0	EKV04334
PC-04	5	492	Conserved PWI domain	*P. chrysogenum*	8e^−132^	XP_002563178
PC-05	6	335	Cytochrome P450 monooxygenase (GA14 Synthase)	*A. oryzae* RIB40	2e^−149^	XP_001827555
PC-06	4	335	NmrA-family transcriptional regulator	*A. flavus* NRRL3357	7e^−120^	XP_002384043
**PC-07 (*penP*)**	**6**	**520**	**Cytochrome P450 monooxygenase**	*P. paxilli*	0.0	AAK11528
**PC-08 (*penC*)**	**3**	**342**	**Prenyl transferase**	*P. chrysogenum*	2e^−168^	XP_002562743
**PC-09 (*penB*)**	**2**	**243**	**Integral membrane protein**	*P. paxilli*	5e^−130^	ADO29934
**PC-10 (*penM*)**	**4**	**465**	**FAD-dependent monooxygenase**	*P. paxilli*	0.0	AAK11530
**PC-11 (*penQ*)**	**9**	**510**	**Cytochrome P450 monooxygenase**	*P. paxilli*	0.0	AAK11527
**PC-12 (*penA*)**	**2**	**368**	**Integral membrane protein**	*P. paxilli*	2e^−53^	ADO29933
**PC-13 (*penD*)**	**2**	**427**	**Aromatic prenyl transferase**	*P. paxilli*	3e^−85^	AAAK11526
PC-14	5	577	Dimethylaniline monooxygenase	*Metarhizium acridum*	2e^−170^	EFY85025
PC-15	3	271	Oxidoreductase/short chain dehydrogenase	*Glarea lozoyensis*	6e^−77^	EPE26761
PC-16	1	494	Acetyltransferase	*A. oryzae* RIB40	5e^−116^	XP_001822009
PC-17	5	543	MFS transporter	*P. chrysogenum*	0.0	XP_002562735
**PC-18 (*penG*)**	**4**	**341**	**Geranylgeranyl diphosphate synthase**	*A. niger* ATCC1015	1e^−133^	EHA20968
**PC-19 (*penO*)**	**4**	**450**	**FAD-binding oxidoreductase**	*A. oryzae* RIB40	1e^−108^	XP_001817261
PC-20	4	453	Cytochrome P450 monooxygenase	*Pyrenophora tritici-repentis*	5e^−49^	XP_001938007
PC-21	3	510	Cytochrome P450 monooxygenase	*Exophiala dermatitidis*	1e^−119^	EHY54727
PC-22	2	425	Aromatic prenyl transferase	*Aspergillus* sp. MF297	1 e^−78^	ADM34132
PC-23	3	204	Cytochrome P450 monooxygenase (sterigmatocystin *stcS*)	*A. flavus* NRRL3357	1e^−37^	XP_002384465

Proposed penitremane biosynthetic genes are shown in bold type.

**Table 2:** Known and predicted functions of genes at the *P. janthinellum JAN* locus.

Gene/ORF	No. of exons	Predicted product size (aa)	Predicted function	Top BLASTp match		
				Organism	*E*-value	Accession no.
PJ-01	6	2361	Pfs domain protein	*Aspergillus fumigatus*	0.0	XP_748404
PJ-02	2	476	Transcriptional regulator Ngg1	*Penicillium oxalicum*	0.0	EPS27964
PJ-03	2	500	RING finger domain protein	*P. chrysogenum*	5e^−100^	XP_002561235
PJ-04	3	203	60S ribosomal protein	*P. oxalicum*	1e^−146^	EPS27962
PJ-05	3	1216	Ubiquitin protein lyase	*P. oxalicum*	0.0	EPS27961
PJ-06	7	800	Beta-glucosidase	*P. oxalicum*	0.0	EPS27960
**PJ-07 *(janG)***	**4**	**312**	**Geranylgeranyl diphosphate synthase**	*P. chrysogenum*	9e^−171^	XP_002562745
**PJ-08 *(janA)***	**2**	**349**	**Integral membrane protein**	*P. paxilli*	4e^−104^	ADO29933
**PJ-09 *(janM)***	**3**	**463**	**FAD-dependent monooxygenase**	*P. paxilli*	0.0	AAK11530
**PJ-10 *(janB)***	**2**	**243**	**Integral membrane protein**	*P. paxilli*	2e^−134^	ADO229934
**PJ-11 *(janC)***	**3**	**327**	**Prenyl transferase**	*P. chrysogenum*	0.0	XP_002562743
**PJ-12 *(janP)***	**6**	**515**	**Cytochrome P450 monooxygenase**	*P. paxilli*	0.0	AAK11528
PJ-13	3	566	Cytochrome P450 monooxygenase	*Ajellomyces dermatitidis*	7e^−128^	EEQ86297
**PJ-14 *(janQ)***	**9**	**469**	**Cytochrome P450 monooxygenase**	*P. paxilli*	0.0	AAK11527
**PJ-15 *(janD)***	**2**	**438**	**Aromatic prenyl transferase**	*P. paxilli*	0.0	AAK11526
**PJ-16 *(janO)***	**4**	**448**	**FAD-binding oxidoreductase**	*P. paxilli*	0.0	ADO29935
PJ-17	4	283	Conserved hypothetical	*Talaromyces marneffei*	6e^−32^	XP_002147239
PJ-18	2	348	Alcohol dehydrogenase	*A. terreus*	0.0	XP_001212944
PJ-19	2	272	FRG1-like family protein	*P. oxalicum*	0.0	EPS27959
PJ-20	3	720	Conserved hypothetical	*P. oxalicum*	0.0	EPS27958
PJ-21	2	514	Glucoronyl hydrolase	*P. oxalicum*	0.0	EPS27957
PJ-22	7	801	Transcriptional regulator	*P. oxalicum*	0.0	EPS27956

Proposed janthitremane biosynthetic genes are shown in bold type.

### 2.2. Bioinformatic Analysis of Indole-Diterpene Cluster Sequences

For *P. crustosum*, 23 predicted open reading frames were identified within the contiguous sequence and designated PC-01 to PC-23 (Figure 2a, Table 1). Nine of these were putative orthologs of genes in the *PAX* cluster. All seven of the genes that are necessary for paxilline biosynthesis in *P. paxilli* [*paxG* (geranygeranyl diphosphate synthase), *paxA* (integral membrane protein), *paxM* (FAD-dependent monooxygenase), *paxB* (integral membrane protein) *paxC* (prenyl transferase) *paxP* (cytochrome P450 monooxygenase) and *paxQ* (cytochrome P450 monooxygenase)], had homologs in the *PEN* cluster plus homologs of two genes [*paxD* (aromatic prenyl transferase) and *paxO* (oxidoreductase)] that have demonstrated or possible functions in paxilline modification [11]. These genes were tandemly arranged in the cluster from PC-07 to PC-13 and PC-18 to PC-19, interrupted by genes PC-14 to PC-17.

These four genes are predicted to encode a putative dimethylaniline monooxygenase (PC-14), an oxidoreductase/short chain dehydrogenase (PC-15), an acetyl transferase (PC-16), and an MFS transporter (PC-17). The *pen* genes were flanked by genes predicted to encode a cytochrome P450 monooxygenase similar to a gene for gibberellin biosynthesis in *F. fujikuroi* (PC-05) [27] and a Nmr-A family transcriptional regulator (PC-06) at one flank and two cytochrome P450s (PC-20 and PC-21) and an aromatic prenyl transferase (PC-22) at the other.

For *P. janthinellum*, 22 predicted open reading frames were identified and designated PJ-01 to PJ-22 (Figure 2b, Table 2). The *JAN* cluster also contained homologs of the nine *pax* genes with identical syntenic organization compared with the *PAX* cluster from PJ-07 to PJ-16, except that these genes were interrupted by a single gene encoding a cytochrome P450 monooxygenase (PJ-13) that does not have a homolog in any other indole-diterpene gene cluster characterized to date. The *jan* genes were flanked by genes predicted to encode a ubiquitin protein ligase and a β-glucosidase (PJ-05 and PJ-06) at one flank, and a hypothetical protein and an alcohol dehydrogenase (PJ-17 and PJ-18) at the other.

Identities with Pax proteins ranged from 31% to 75% for *P. crustosum* and 42% to 77% for *P. janthinellum*. All of the *pen* and *jan* genes were predicted to contain multiple exons as shown in Table 1 and Table 2 and displayed a high level of structural conservation compared with the *pax* genes in terms of the positions of introns within their coding sequences. Exceptions to this were for *penM*, which had one additional intron compared with *paxM*, and *penQ* which had one less intron than *paxQ*. Barring these exceptions, of 27 introns identified in the nine *pax* genes of *P. paxilli*, equivalent introns were identified in the same relative positions in the respective *pen* and *jan* gene homologs of *P. crustosum* and *P. janthinellum*.

Core genes for the initial stages of indole-diterpene biosynthesis in *P. paxilli* are *paxG*, *paxC* and *paxM*. The *P. crustosum* and *P. janthinellum* homologs of these genes identified in the *PEN* and *JAN* clusters (Table 1 and Table 2), namely *penG*/*janG*, *penC*/*janC*, and *penM*/*janM* are each predicted to encode a generanyl geranyl diphosphate synthase, a prenyl transferase and a FAD-dependent monooxygenase with 49%/42%, 38%/71%, and 61%/67% amino acid sequence identity compared with their respective Pax homologues. The predicted protein products of genes *penA*/*janA* and *penB*/*janB* are homologs of PaxA and PaxB. PaxB, an integral membrane protein, together with PaxM are proposed to catalyse epoxidation and cyclisation of the diterpene skeleton for paspaline biosynthesis [13,14]. PaxA is also a putative integral membrane protein but its precise role in indole-diterpene biosynthesis has yet to be determined. PenA/JanA and PenB/JanB shared 38%/50% and 72%/77% sequence identity compared with PaxA and PaxB, respectively. Genes *paxP* and *paxQ* that encode cytochrome P450 monooxygenases in *P. paxilli* [12,15] complete the collection of seven genes required for paxilline biosynthesis in this fungus. Homologs identified in the *PEN* and *JAN* clusters (*penP*/*janP* and *penQ*/*janQ*) had amino acid identities of 75%/71% and 51%/65%, respectively. These predicted proteins both contained all of the functional domains expected for cytochrome P450s including heme-binding domains identical to those of PaxP and PaxQ (H**FG**L**G**RYA**C** for the PaxP-like proteins, and Q**FG**D**G**RHT**C** for PaxQ-like proteins) [12,28]. Ancillary genes whose protein products are likely to have a subsequent role in the modification of the indole-diterpene core are *penD*/*janD*, and *penO*/*janO*. These genes are predicted to encode aromatic prenyltransferases and FAD-binding oxidoreductases, and compared with their Pax homologues had 31%/66% and 41%/72% amino acid identity, respectively.

### 2.3. Disruption of Indole-Diterpene Biosynthesis by Deletion of Genes in the PEN and JAN Clusters of P. crustosum and P. janthinellum

**Figure 3:**
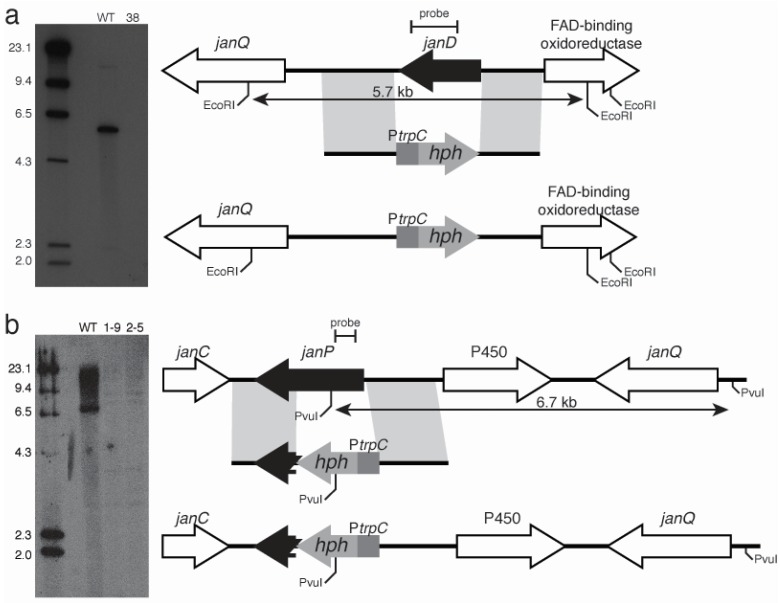

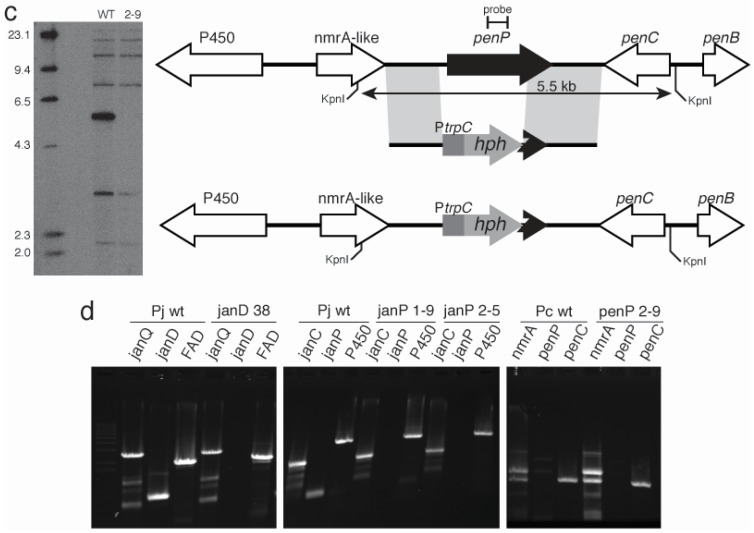
Indole-diterpene biosynthesis gene replacements for (**a**) *janD* and (**b**) *janP* at the *JAN* locus in *P. janthinellum* (mutants #38, and #1-9 and #2-5, respectively), and (**c**) *penP* at the *PEN* locus in *P. crustosum* (mutant #2-9). Physical maps of the respective genomic regions with linear replacement constructs and mutant alleles with autoradiographs of Southern blots of 1 μg genomic digest probed with [^32^P]-dCTP-labelled fragments from within the deleted region of each gene. Expected bands in the Southern analyses were 5.7-kb for *janD* (**a**) 6.7-kb for *janP* (**b**) 5.5-kb for *penP* (**c**). (**d**) PCR amplification analysis of targeted and flanking genes from wild-type and mutant strains using primers described in Table A2.

To demonstrate involvement of genes from the *PEN* and *JAN* clusters in indole-diterpene biosynthesis gene replacement constructs pCE50, pCE51, and pCS12 were prepared to enable targeted deletion of *penP*, *janP*, and *janD*, respectively. Successful gene replacements were confirmed by Southern and PCR analysis of hygromycin-resistant transformants (Figure 3).

**Figure 4:**
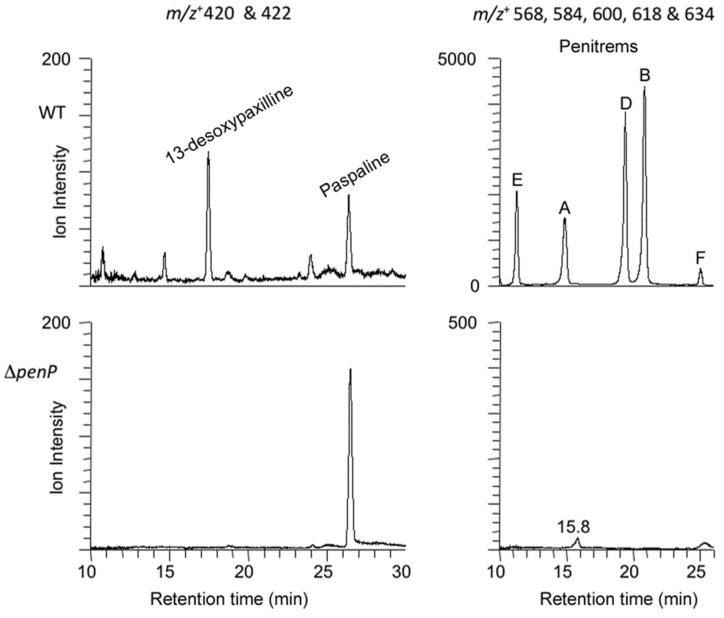
Extracted ion chromatograms for *P. crustosum* wild-type (WT) and ∆*penP* deletion mutant showing LC-MS peaks for 13-desoxypaxilline (17.5 min, *m/z* + 420), paspaline (26.4 min, *m/z* + 422) and penitrems E, A, D, B, and F (respectively 11.3 min, *m/z* + 568; 14.9 min, *m/z* + 584; 19.4 min, *m/z* + 600; 20.8 min, *m/z* + 618; and 25.0 min, *m/z* + 634).

LC-MS analysis showed that the ∆*penP* mutant of *P. crustosum* did not produce any of the penitrems or 13-desoxypaxilline that were present in the wild-type strain but instead accumulated the early pathway intermediate paspaline as shown in Figure 4.

**Figure 5:**
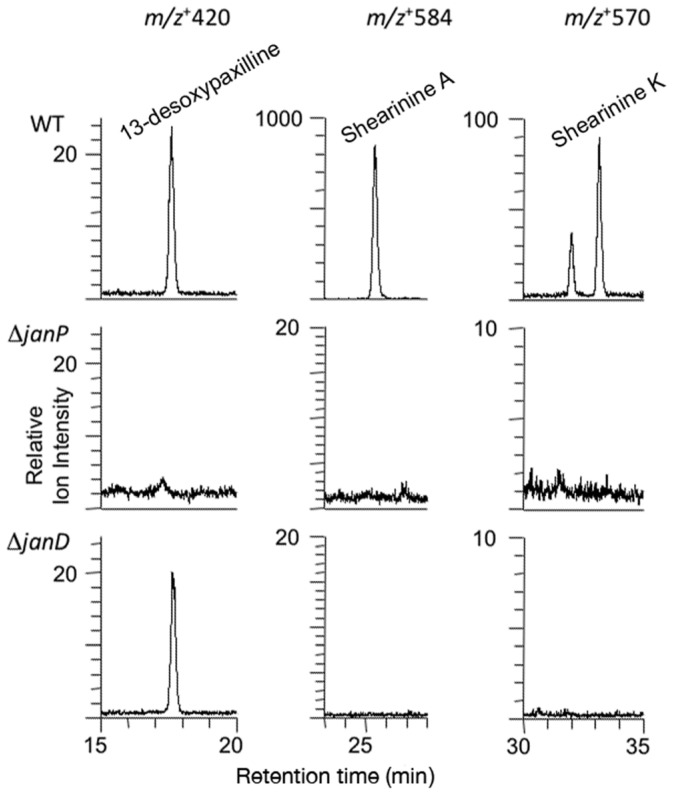
Extracted ion chromatograms for *P. janthinellum* wild-type (WT) and ∆*janP* and ∆*janD* deletion mutants showing LC-MS peaks for 13-desoxypaxilline (17.6 min, *m*/*z* + 420), and prenyl-elaborated indole-diterpenes (25.5 min, *m*/*z* + 584 attributable to shearinine A or F; 32.0 min and 33.2 min, both *m*/*z* + 570 attributable to shearinine K or isomers).

The *P. janthinellum* wild type strain produced 13-desoxypaxilline and prenyl-elaborated indole-diterpenes of higher mass, *m/z*+ 584 (attributable to the janthitremanes shearinine A or F) and *m/z* + 570 (attributable to aflatremane diprenyl-paspalinines such as shearinine K or its isomers) [24,29,30]. However, no indole-diterpenes were identified in the ∆*janP* mutant and only increased 13-desoxypaxilline was detected in the ∆*janD* mutant as shown in Figure 5. An MS/MS fragment ion of *m/z* + 222 observed coincident with the parent *m/z* + 584 compound is evidence in support of the janthitremane attribution, this fragmentation having been previously observed in the MS/MS analysis of other structurally related janthitremanes. Possible trace amounts of paspaline were insufficient for secure identification.

## 3. Discussion

We describe here the cloning and molecular analysis of two new indole-diterpene gene clusters from *P. crustosum* and *P. janthinellum*, with genes that encode enzymes for the synthesis of penitrems and shearinines, respectively. Both clusters contain homologs of the core set of four genes, *paxGMBC*, shown to be required for paspaline biosynthesis in *P. paxilli* [13], and that are present in all indole-diterpene gene clusters characterized to date [17,19], supporting the hypothesis that paspaline is the core cyclic intermediate for the synthesis of a range of indole-diterpenes. In addition, both clusters have homologs of *paxP* (*penP*/*janP*) and *paxQ* (*penQ*/*janQ*), which encode multifunctional cytochrome P450 moonoxygenases for key post-paspaline biosynthetic steps [15], as well as homologs of *paxA*, which encodes an integral membrane protein of unknown function, *paxD*, encoding a dimethylallyl transferase [11], and *paxO*, encoding an oxido-reductase that is probably involved in cyclisation of prenylated paxilline.

Both the order and the orientation of these nine genes in the *P. janithenllum jan* cluster are identical to the corresponding nine *pax* genes in *P. paxilli*. However, the one distinct difference between these two clusters is the presence of an extra gene encoding a cytochrome P450 monooxygenase, PJ-13, between *janP* and *janQ*. The predicted functions of the genes adjacent to the left- (PJ-05 encoding a ubiquitin ligase and PJ-06 encoding a β-glucosidase) and the right- (PJ-17 encoding a hypothetical protein and PJ-18 encoding an alcohol dehydrogenase) flanks of this cluster would suggest these additional genes have no role in the biosynthesis of janthitremanes. The absence of detectable levels of diprenyl-elaborated indole-diterpenes (including proposed shearinines K and A) in the *janP* and *janD* deletion mutants of *P. janthinellum* and accumulation of the intermediate 13-desoxypaxilline in the latter, provide genetic evidence that this gene cluster is responsible for janthitremane biosynthesis. Our inability to detect paspaline in the *janP* mutant was surprising but may be associated with the difficulty we have encountered in readily inducing sporulation and secondary metabolite biosynthesis in this particular *P. janthinellum* strain.

**Figure 6:**
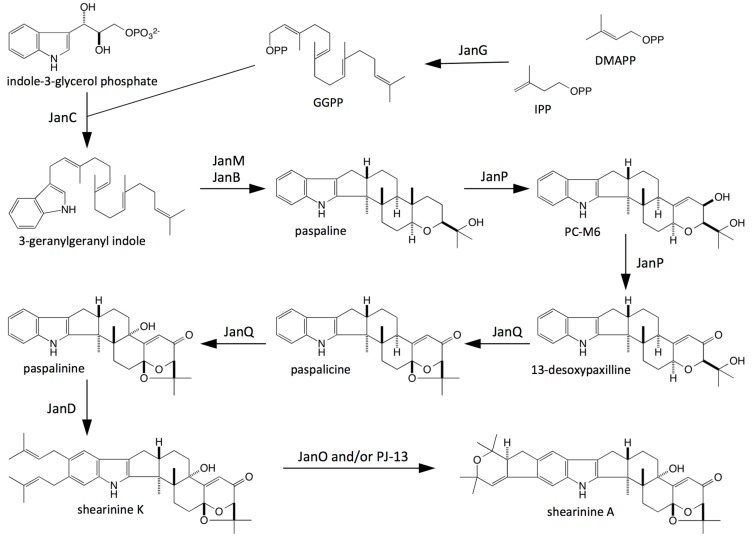
Proposed biosynthetic scheme for shearinine K and A biosynthesis in *P. janthinellum*.

By analogy with the known pathway for paxilline biosynthesis in *P. paxilli* [11,14], heterologous functional analysis of key biosynthetic steps in aflatrem biosynthesis in *A. flavus* [19,31], and a comparison of the structures of the main products of these two pathways with the structures of shearinines A and K, a proposed pathway for the synthesis of these compounds in *P. janithinellum* is presented in Figure 6. This scheme proposes that JanG catalyses the synthesis of geranygeranyl diphosphate (GGPP), which condenses with indole 3-glycerol phosphate to form 3-geranylgeranylindole (GG-I) in the presence of JanC, followed by epoxidation and cyclisation steps catalyzed by JanM and JanB, to form paspaline. JanP is proposed to catalyse the conversion of paspapline to 13-desoxypaspaline via β-PC-M6 in a series of α-face oxidations as occurs in both *P. paxilli* and *A. flavus* [15,19,31].

Like AtmQ from *A. flavus*, JanQ is proposed to carry out sequential β-face oxidation steps at C-7 and C-13 of 13-desoxypaspaline to form paspalicine and paspalinine respectively. By analogy to the diprenylation of the indole ring of paxilline by PaxD, JanD is proposed to carry out a similar reaction in *P. janthinellum* to form shearinine K [11,24,30,32]. Further oxidation and cyclisation of this compound by JanO and/or PJ-13 would generate shearinine A [29,30].

In contrast to the janthitremanes, the penitremanes are chemically much more complex and would therefore require more enzymes for their synthesis. The gene content and organization of the *P. crustosum pen* cluster is correspondingly much more complex than the *P. janthinellum jan* cluster. While homologs of the nine genes identified in *P. paxilli* and *P. janthinellum* were found in this cluster, a number of additional genes encoding enzymes with functions likely to be required for penitremane biosynthesis were identified in this cluster including: PC-05, PC-20, PC-21, and PC-23 encoding cytochrome P450 monooxygenases and PC-22 encoding a dimethylallyl prenyl transferase. Both the order and the orientation of all the genes in this *P. crustosum* cluster from PC-05 through to PC-23 (19 genes) are identical to a cluster of genes recently identified in *P. simplicissimum* required for penitrem biosynthesis [33]. The absence of detectable levels of penitrems A, B, D, E, and F, and 13-desoxypaxilline in the *penP* deletion mutant of *P. crustosum*, but instead an accumulation of paspaline, provides genetic evidence that this gene cluster is responsible for penitrem biosynthesis.

In a *tour de force* Liu *et al.* [33] recently succeeded in elucidating the function of 17 of the proposed 20 genes required for penitrem biosynthesis in *P. simplicissimum* by heterologous expression of blocks of these genes in *A. oryzae*. On the basis of these reconstitution experiments they propose that PtmGCMB (PenGCMB) are responsible for the synthesis of paspaline and PtmPQ (PenPQ) for subsequent conversion to paxilline, which serves as a substrate for a three step process consisting of C10 ketoreduction (PtmH/PC-15), followed by C20 prenylation (PtmD/PenD) and dehydration (PtmV/PC-16, PtmI/not annotated) to generate the dehydration product of mono-prenylated β-paxitriol. This key intermediate is then converted to PC-M4 via the action of an oxidoreductase (PtmO/PenO) and a second dimethylallyl prenyl transferase (PtmE/PC-22). A series of oxidation steps involving 4 cytochrome P450 monooxygenases (PtmK, PtmU, PtmL, PtmJ/PC-21, PC-05, PC-23, PC-20) and an FAD-dependent monooxygenase (PtmN/PC-14) are required for the transformation of PC-M4 to penitrems A and E. Synthesis of these final products is proposed to proceed via penitrems D and C (PtmK, PtmU, PtmN/PC-21, PC-05, PC-14) and penitrems B and F (PtmK, PtmU, PtmN, PtmL/PC-21, PC-05, PC-14, PC-23). This analysis and earlier work demonstrates the power of the *A. oryzae* heterologous gene expression system for biological synthesis of complex natural products [14,31,33].

In summary, the work described here and in earlier papers provides a genetic basis for the diversity of indole-diterpene natural products found within the *Penicillium* and *Aspergillus* species. All the indole-diterpene gene clusters identified to date have a core set of genes for the synthesis of paspaline, and a suite of additional genes that encode multi-functional cytochrome P450 monooxygenases, FAD dependent monooxygenases and prenyl transferases that catalyse various regio- and stereo- specific oxidations on this molecular skeleton to generate a diversity of indole-diterpene products.

## 4. Experimental Section

### 4.1. Fungal Strains and Growth Conditions

Cultures of wild-type *P. crustosum* (isolate PN2402) and *P. janthinellum* (isolate PN2408) were maintained on 2.4% Difco potato dextrose agar (Beckton Dickinson, MD, USA) plates or as spore suspensions in 10% (*v*/*v*) glycerol at −80 °C (Table A3). For indole-diterpene production, 100 mL Erlenmeyer flasks containing 25 mL of YEPGA medium [12] were inoculated with 5 × 10^6^ spores and grown shaking (200 rpm) for 48 h at 28 °C. Aliquots of 2 mL were used to inoculate 250 mL Erlenmeyer flasks containing 50 mL of aflatrem production medium [18] which were grown without agitation at 29 °C in the dark for ten days. The mycelial mat that formed on the surface of the liquid was harvested, washed in Milli-Q water (Millipore, MA, USA) and freeze-dried for indole-diterpene analysis. Fungal samples were stored at −80 °C prior to drying or analysis. For isolation of genomic DNA, 25 mL of CDYE medium [10] was inoculated with 5 × 10^6^ spores and grown shaking (200 rpm) for 48 h at 30 °C. Mycelium was harvested, washed in Milli-Q water (Millipore), and freeze dried.

### 4.2. Isolation, PCR-Amplification, and Sequencing of Genomic DNA

Genomic DNA was isolated from freeze-dried mycelia using the method of Yoder [34] after grinding in liquid nitrogen with a pestle and mortar. Genomic DNA was amplified using the TripleMaster PCR system (Eppendorf, Hamburg, Germany). Reactions were each performed in a 50 µL volume that contained 1 × High-Fidelity buffer with final concentrations of 4 mM magnesium acetate, 200 µM of each dNTP, 2 U TripleMaster polymerase mixture, 400 nM of each primer, and 50 ng of genomic DNA. Thermal cycling was performed in a Mastercycle gradient thermocycler (Eppendorf) with the following conditions: two min at 94 °C followed by 30 cycles of 94 °C for 30 sec, 65 °C for 30 sec, and 72 °C for 3 min; with a final elongation for 10 min at 72 °C. Primers used for amplification of indole-diterpene cluster sequences are shown in Table A1. PCR products were purified using the Wizard SV Gel and PCR Clean-Up System (Promega, Madison, WI, USA) prior to sequencing. At least two independent PCR reactions were combined and sequenced directly on both strands using the dideoxynucleotide chain termination method with the Big-Dye Terminator (version 3.1) Cycle Sequencing Kit (Applied Biosystems, Foster City, CA, USA) and separated using an ABI3730 Genetic Analyzer (Applied Biosystems).

### 4.3. Cosmid Library Production and Screening

Fungal protoplasts were prepared as described previously [12] except that *P. crustosum* cultures were grown shaking for 21 h and *P. janthinellum* for 30 h. High molecular weight genomic DNA isolated from protoplasts using the method of Byrd *et al.* [35] was partially digested using *Mbo*I in a reaction containing 7.5 µg gDNA and enzyme concentrations of 0.141 U and 0.126 U per µg gDNA for *P. crustosum* and *P. janthinellum*, respectively. Reaction volumes of 100 µL were incubated at 37 °C for 60 min. Partially digested gDNA was end-filled with dATP and dGTP to generate 5'-GA protruding termini. Cosmid vector pMOcosX (120 µg) [36] was digested with 100 U each of *Xba*I and *Xho*I in a final reaction volume of 300 µL for 2 h at 37 °C to yield left and right cosmid arms. Purified digested cosmid DNA was partially end-filled with dTTP and dCTP to generate protruding 5'-TC termini. End filling reactions were performed in 100 µL reaction volumes containing 4 µg gDNA or 20 µg cosmid DNA with 1 U/µg of the Klenow fragment of DNA polymerase I (Invitrogen, CA, USA) and 0.5 mM of each of dTTP and dCTP (for cosmid DNA) or dATP and dGTP (for gDNA) incubated at room temperature for 20 min. Digestion and end-filling reactions were each stopped and purified by phenol:chloroform extraction according to standard molecular biology techniques [37] and resuspended in 50 µL MilliQ water.

Ligation reactions containing digested and end-filled cosmid DNA (6 µg) and gDNA (1.5 µg) in final volumes of 60 µL containing 9 Weiss units of T4 DNA ligase (Promega) were incubated at 16 °C for 6 h then stored at 4 °C. Packaging reactions and transduction of host cells were performed using Gigapack III Gold Packaging Extract and *E. coli* host strain VCS257, respectively according to the manufacturer’s recommendations (Stratagene, La Jolla, CA, USA). Libraries were amplified and for each, approximately 12,000 ampicillin-resistant transformants were spread on six 90 mm diameter agar plates. The libraries were screened using radioactively labelled hybridization probes according to standard molecular biology techniques [37]. The *P. crustosum* library was screened using a 1448-bp PCR fragment amplified using primers 2402CF1 and 2402PF1 for *P. janthinellum* PN2408 a 1045-bp fragment amplified using primers 2408F1 and 2408PF1 (Table A1) which contained the 3' ends of *paxC* and *paxP* homologs and corresponding intergenic regions, respectively.

### 4.4. Cosmid Sequencing

The Illumina Genome Analyzer IIx was used to generate 75-bp paired-end reads, which were processed with version 1.6 of Illumina’s data analysis pipeline. Reads were trimmed to the largest contiguous sequence where quality scores exceeded *P* = 0.05 [38]. After trimming, reads shorter than 25 bp were discarded. Reads were *de novo* assembled using the de Bruijn graph assembler *ABySS* (version 1.2.0) [39]. Trimmed and untrimmed reads were assembled separately using a range of *k*-mer values [19–61, odd numbers only] and a parameter sweep of *n* [2,5,10], *c* [1,10,20] and *e* [1,3,10]. The assembly with the most complete coverage of the cosmid, as recognized from flanking vector sequence, was extracted manually. Gaps in the assembly were corrected manually by patching with sequence information from other assemblies.

### 4.5. Bioinformatics

Database searches were performed at the National Center for Biotechnology Information website using BLASTx against the nucleotide collection database and BLASTp against the non-redundant protein and SWISSPROT databases. Subsequent gene predictions were made using FGENESH gene finding program with organism-specific gene-finding parameters for *Aspergillus nidulans* [40]. Identity and similarity scores were calculated after ClustalW alignment of sequences using MacVector version 9.5 (MacVector Inc.).

### 4.6. Preparation of Gene Replacement Constructs and Southern Analysis

The *penP*, *janP,* and *janD* replacement constructs pCE50, pCE51, and pCS5 were prepared by yeast recombination cloning [41] using *Eco*RI/*Xho*I cut pRS426, 5' and 3' fragments to each of the genes amplified from genomic DNA using Phusion™ High-Fidelity DNA polymerase (Thermo Scientific, Waltham, MA, USA), together with a 1.38-kb *PtrpC-hph* fragment amplified from pSF15.15 using primer pair hph-F/hph-R (Table A4). The 921-bp 5' and 1271-bp 3' fragments of *penP* were amplified using primer pairs pRS426-penP-F/penP-hph-R and hph-penP-F/penP-pRS426-R, respectively. The 1152-bp 5' and 1071-bp 3' fragments of *janP* were amplified using primer pairs pRS426-janP-F/janP-hph-R and hph-janP-F/janP-pRS426-R, respectively. The 1205-bp 5' and 1002-bp 3' fragments of *janD* were amplified using primer pairs CSPjantD1F/CSPjantD2R and CSPjantD3F/CSPjantD4R, respectively.

To facilitate yeast recombinational cloning, primers for amplification of the *penP*, *janP,* and *janD* 5' flanking fragments contained overlap to the yeast vector pRS426 (pRS426-penP-F, pRS426-janP-F and CSPjantD1F) and to the *hph* hygromycin resistance cassette (penP-hph-R, janP-hph-R and CSPjantD2R), and primers for amplification of the 3' flanking fragment contained overlap to the *hph* resistance cassette (hph-penP-F, hph-janP-F and CSPjantD3F) and to pRS426 (penP-pRS426-R, janP-pRS426-R and CSPjantD4R).

Yeast cells were then transformed with *Eco*RI/*Xho*I linearised pRS426, PCR amplified P*trpC*-*hph* cassette, and *penP*, *janP* or *janD* 5' and 3' flanking region PCR fragments as previously described [42]. Transformants were selected on media lacking uracil and plasmid DNA subsequently isolated and transformed into *E. coli*. Plasmid DNA was isolated from resulting *E. coli* transformants and analysed for the correct construct by diagnostic PCR and DNA sequencing.

Lists of all plasmids and the primer sequences used to prepare those constructs can be found in Table A3 and Table A4.

### 4.7. Fungal Transformation

Protoplasts of wild-type *P. crustosum* and *P. janthinellum* were prepared and transformed as previously described for *P. paxilli* [13]. For replacement of *P. crustosum penP*, protoplasts were transformed with 5 μg of linear PCR product amplified with Phusion™ High-Fidelity DNA polymerase using pCE50 as template with primer set pRS426-penP-F and penP-pRS426-R. For replacement of *P. janthinellum janP* and *janD* protoplasts were transformed with 5 μg of linear PCR product amplified with Phusion™ High-Fidelity DNA polymerase using pCE51 and pCS5 as template with primer sets pRS426-janP-F and janP-pRS426-R, and CSPjantD1F and CSPjantD4R, respectively. *P. crustosum* and *P. janthinellum* transformants were selected on regeneration medium supplemented with 200- and 250-μg/mL hygromycin respectively (400 μg/mL for *janD* deletions). Southern blotting, probe labeling, and hybridization were carried out as previously described [16].

### 4.8. Indole-Diterpene Chemical Analysis

Freeze dried fungal biomass (0.5 or 1 g) was homogenized in 25 mL of 7:3 (*v*/*v*) propan-2-ol-water mixture at ambient temperature for extraction of the indole-diterpenes. After mixing for 1 h, the samples were centrifuged to pellet the insoluble residue. The supernatants were stored at −18 °C.

LC-MS/MS analysis was performed using a system consisting of a Thermo PAL sampler, Thermo Accela pump, and Thermo LTQ XL linear ion trap mass spectrometer in ESI positive ion mode (Thermo Scientific, Waltham, CA, USA). A Luna C18 column (150 × 2 mm, 5 µm); Phenomenex, Torrance, CA, USA) was used for separations at a flow rate of 200 µL/min of a linear gradient over 40 min, beginning at 1:1 acetonitrile-water with 0.1% (*v*/*v*) acetic acid through to acetonitrile with 0.1% acetic acid, this held for a further 20 min. The mass spectrometer was setup in data-dependent mode for collection of fragmentation data of a range of preselected ions to assist attribution of identities of indole-diterpene compounds.

### 4.9. Nucleotide Sequence Accession Numbers

Contiguous sequences containing the *PEN* cluster from *P. crustosum* and *JAN* cluster from *P. janthinellum* have been deposited with GenBank under accession numbers KC963408 and KF280651, respectively.

## Appendix

**Table A1:** Degenerate and gene-specific oligonucleotide primers used for PCR amplification of indole-diterpene biosynthesis gene cluster fragments from *P. crustosum* and *P. janthinellum*.

Primer 1		Primer 2		Target	Product (Species & Size)
Name	Sequence	Name	Sequence		
ConC1	TGCTTGATGATGGTCGAYGAT	ConC2	GACATTCTTGCAGTCATTTTG	paxC homologs	*P. crust*. 529-bp *P. janth*. 53-bp
2402F1	GGATGATACCATGACCCTTGTCGCGTAT	PBR2	TANGCNARNGCNGGNCCDATYTC	P. crust. C to P	*P. crust*. 1611-bp
		2402PF1	CGGTTCTTCGCTGCCTTCGTCATGAAG	P. crust. C to P	*P. crust*. 1448-bp (probe)
PPF1	AARCMNGAYGAYTTYYTNCARTGG	PPR2	CANGCRTANCKNCCNARNCCRAARTG	paxP homologs	*P. janth*. 601-bp
2408F1	CTTTACCGTGATTGCGTAAGTCGCATT	2408PF1	GTGAGTACGGGTCCAAGTCATATGCAT	P. janth. C to P	1045-bp (probe)

**Table A2:** Primers used for screening gene deletions.

Primer Name	Sequence	Gene
CSnmrAF	AGTAGGGTAGGCAGCATCCA	Check for PC-06 (NmrA)
CSnmrAR	GCGCACTATTTCTGAGCAGC	Check for PC-06
pen1	ATGTCCAGATCCCTAACCCAC	Screen for penP deletion
pen2	CATAATTGGGGTCACCTGATG	Screen for penP deletion
CSpenCF	CGTCGACAGAATCTCCAGCT	Check for penC
CSpenCR	TCATCCATCACACCGCGATT	Check for penC
CSjanCF	GGGGAAGGTAGCCATGCTTT	Check for janC
CSjanCR	TGCGGATATTACGAGGCGAC	Check for janC
jan1	CCAATGTTAGAGTCCCAACG	Screen for jan P deletion
jan2	GTCGTATACTCCCCGACATG	Screen for jan P deletion
CSP450F	ACGGTCACGGTCAAGTCTTC	Check for PJ-13 (P450)
CSP450R	CGTTACAGGGCCGGGTATTT	Check for PJ-13 (P450)
CSjanQF	TACAGGCCAGCTCTTCAACC	Check for janQ
CSjanQR	TCGGACATCTTTCGCACCAA	Check for janQ
CS janD intF	GGACTTACAAGGCTTTCGCA	Screen for janD deletion
CS janD intR	ACGCTTAAAGCCCAGAAACA	Screen for janD deletion
CSFADF	CTTCCTCGTGCCCTTGACAT	Check for janO (FAD)
CSFADR	GACCATGCTGTACCGAACGT	Check for janO (FAD)

**Table A3:** Biological material.

Biological Material	Relevant Characteristics	Reference
**Fungal strains**		
*Penicillium crustosum*		
PN2402 (T2/3013)	Wild-type	Margaret di Menna
PN2897 (CE68/∆*penP*#2-9)	PN2402/∆*penP*::P*trpC*-*hph*; Hyg^R^	This study
*Penicillium janthinellum*		
PN2408 (C1P3)	Wild-type	Margaret di Menna
PN2898 (CE71/∆*janP*#1-9)	PN2408/∆*janP*::P*trpC*-*hph*; Hyg^R^	This study
PN2899 (CE70/∆*janP*#2-5)	PN2408/∆*janP*::P*trpC*-*hph*; Hyg^R^	This study
PN2883 (∆*janD*#38)	PN2408/∆*janD*::P*trpC*-*hph*; Hyg^R^	This study
**Yeast strains**		
*Saccharomyces cerevisiae*		
FY834 (PN2806)	*MATa his3* 200ð *ura3-52 leu2 1 lys2 202 trp1 63*	[43]
**Bacterial strains**		
*Escherichia coli*		
DH5α	F^−^, ϕ80*lacZ*, DM15, ∆(*lacZYA*-*argF*), U169, *recA1*, *endA1*, *hsdR17* (r_k_^−^, m_k_^−^), *phoA*, *supE44*, λ^−^, *thi-1*, *gyrA96*, *relA1*	Invitrogen
VCS257	*supE44 supF58 hsdS3*(r_B_^−^ m_B_^−^) *dapD8 lacY1 glnV44* Δ(*gal-uvrB*)*47 tyrT58 gyrA29 tonA53* Δ(*thyA57*)	Stratagene
PN4138	DH5α/pRS426	
PN4139	DH5α/pCE50 penP1	This study
PN4140	DH5α/pCE51 janP1	This study
PN4142	DH5α/pCE52 janP2	This study
PN4172	DH5α/pCS5 janD	This study
**Plasmids**		
pII99 (PN1687)	P*trpC*-*nptII*-T*trpC*, Amp^R^/Gen^R^	[44]
pMOcosX	Amp^R^	[36]
pSF15.15	pSP72 containing 1.4-kb *Hin*dIII P*trpC*-*hph* from pCB1004 cloned into *Sma*I site	[45]
pRS426	ori(f1)-*lacZ*-T7 promoter-MCS (*Kpn*I-*Sac*I)-T3 promoter-*lacI*-ori(pMB1)-ampR-ori (2 micron), *URA3*, Amp^R^	Fungal Genetics Stock Center
pCE50	pRS426 containing 5′*penP*-P*trpC*-*hph*-3′*penP*; Amp^R^/Hyg^R^	This study
pCE51	pRS426 containing 5′*japP*-P*trpC*-*hph*-3′*janP*; Amp^R^/Hyg^R^	This study
pCS5	pRS426 containing 5′*janD*-P*trpC*-*hph*-3′*janD*; Amp^R^/Hyg^R^	This study

**Table A4:** Oligonucleotide primers used for PCR amplification of fragments for creation of gene replacement constructs and screening of transformants.

Primer 1		Primer 2		Target
Name	Sequence	Name	Sequence	
pRS426-penP-F	**GTAACGCCAGGGTTTTCCCAGTCACGAC**AAGCTTTCTGCCCATGTAGCTGCTCAG	penP-hph-R	**ATGCTCCTTCAATATCAGTTCCAAGCT**GCAAGGCGTTGAATCACCCTG	*penP* 5' flank
hph-penP-F	**CCAGCACTCGTCCGAGGGCAAAGGAATAG**ACGCAGAACCTGGCAGTCTCG	penP-pRS426-R	**GCGGATAACAATTTCACACAGGAAACAGC**CTCGAGGGAGATTCTGTCGACGGAATG	*penP* 3' flank
pRS426-janP-F	**GTAACGCCAGGGTTTTCCCAGTCACGAC**GAATTCGCTCGTATCACTTCATAGCAG	janP-hph-R	**AAATGCTCCTTCAATATCAGTTCCAAGCT**GAAGTGGATGGTGTAGGAAGC	*janP* 5' flank
hph-janP-F	**CCAGCACTCGTCCGAGGGCAAAGGAATAG**ACCTGGCTCAACGACTGCTTG	janP-pRS426-R	**GCGGATAACAATTTCACACAGGAAACAGC**CTCGAGTTGCTGGATGGATAGACTTCG	*janP* 3' flank
CSPjantD1F	**GTAACGCCAGGGTTTTCCCAGTCACGAC**ATGCCTTTGTATTAACCGCT	CSPjantD2R	**ATGCTCCTTCAATATCAGTTCCAAGCT**AATCTTCTAGAGACTTTGAGGG	*janD* 5' flank
CSPjantD3F	**CCAGCACTCGTCCGAGGGCAAAGGAATAG**GAAGTGGAAAGATTAGGTTTGGTC	CSPjantD4R	**GCGGATAACAATTTCACACAGGAAACAGC**TCGGGCTTAAATAGATGTCAAGG	*janD* 3' flank
hph-F	AGCTTGGAACTGATATTGAAGG	hph-R	CTATTCCTTTGCCCTCGGACG	P*trpC*-*hph* (hygromycin resistance)

Nucleotides in bold correspond to sequences with homology to adjacent fragment to facilitate yeast recombination.
